# Medicine for global health: can “simple interventions” improve the worldwide burden of disease?

**DOI:** 10.1186/1741-7015-11-72

**Published:** 2013-03-14

**Authors:** Gretchen Birbeck

**Affiliations:** 1 International Neurologic & Psychiatric Epidemiology Program, Michigan State University, 909 Fee Road, East Lansing, MI 48824, USA

## Abstract

Improvements to medical practice and delivery of treatment has been the focus of many international collaborations aiming to address the delivery of appropriate health care in low- and middle-income countries. However, this is compounded by various social, cultural as well as resource allocation issues. This Editorial marks the launch of an article collection on Medicine for Global Health (http://www.biomedcentral.com/bmcmed/series/medicine_for_global_health), and here, guest editor Gretchen Birbeck discusses the challenges, importance and increasing relevance of global health.

## Editorial

“Global health” refers to issues related to population health that, due to the common multi-national nature of the issue or its scientific implications, have relevance beyond a single country’s borders. Given the broad determinants of human health, “global health” topics span a vast spectrum of disciplines compared to with traditional Western medicine. By its very nature, global health must grapple with problems that transcend care provision at the individual patient level. Global health includes ill-defined intersections among human and veterinary medicine (the basic and the clinical), sociology, anthropology, bioethics, environmental health, health services research, economics and the political sciences [1]. Even this list is not exhaustive. Global health is difficult to define—and yet we know it when we see it. Or so our editorial team thinks. We hope you agree.

Much that is published in the global health literature addresses the challenges of public health improvements and healthcare delivery in resource-limited settings. Of course, resources for optimizing health and providing healthcare are virtually always limited in some fashion. But the term “resource-limited setting” is usually used to refer to situations in which the lack of resources is so extreme that the effect of the resource limitation itself becomes one of the central challenges to be assessed. Like the term “*global health*”, the term “*resource-limited setting*” is also amorphous. It can describe a community with food insecurity so severe as to result in increased child mortality or it can be a label used to bemoan the lack of neuroimaging capacity to study cerebral malaria in endemic regions. Dissecting the layers of deprivation and deprivation’s impact on human health in resource-limited settings is one of the many challenges of providing healthcare services and conducting research in such circumstances.

In recent years, the interest in global health from the general public as well as the academic community has skyrocketed. In the first decade of 2000, the number of US medical schools offering international electives more than doubled [2]. Many domestic philanthropic organizations from wealthy nations have expanded into low income, tropical settings [3]. International aid for development assistance was $5.2 billion in 1990, had increased to $21.8 billion by 2007 [3], and even in the face of a global recession, this investment continues to rise. What has stimulated this growing interest? The HIV/AIDS pandemic and subsequent moral imperative to facilitate treatment access beyond wealthy nations undoubtedly drew attention to other healthcare issues that transcend national borders. Philanthropists like Bill and Melinda Gates certainly deserve credit for directing their substantial wealth and personal energies towards highlighting opportunities for improving global health. But perhaps our interest in people and problems beyond our own immediate communities has been most affected by the interconnectedness that characterizes this electronic age.

Simple interventions may be one of the themes of the article collection *Medicine for Global Health*, published in *BMC Medicine.* We learn from a systematic review that hypothermia preventive measures could decrease neonatal mortality rates, since neonatal hypothermia can indirectly contribute to this mortality rate [4]. In another research article, Srinivasan *et al. *[5] report a randomized controlled trial of zinc supplements in children with severe pneumonia. They found an overall case fatality rate of 4% in the zinc-treated children versus 11.9% in those receiving placebo, with the protective effect being greatest for children with HIV. And Hall *et al*. [6] detail the importance of including nutritional support as a key component of any intervention, particularly community-based mass treatment programs, aimed at the neglected tropical diseases (NTDs). The sad reality is that what many clinicians most need to help their patients is the capacity to write a prescription for adequate nutrition. Despite the substantial investments made in the provision of improved access to drugs for conditions including HIV/AIDS, malaria, and NTDs, many of our patients are trying to take these medications on a chronically empty stomach. Maybe “hunger” should be added to the list of NTDs.

Tools developed by the World Health Organization (WHO)-convened expert groups, which are aimed at optimizing healthcare services, have been highlighted in Katchanov’s article on epilepsy care guidelines for low- and middle-income countries [7] as well as the guide for analysts by Jit *et al*. [8] on economic evaluations of the human papillomavirus (HPV) vaccine. HPV cost analyses are the focus of Hutubessy *et al*. [9] and Quentin *et al*. [10], contributions that both detail economic analyses of the costs of delivery and scale-up of the vaccine to girls in Tanzania, where cervical cancer is the number one cancer-related cause of death among women. Taking advantage of planning tools developed by the WHO, Hutubessy *et al*. found that the principal marginal costs are those associated with social mobilization. Accessing girls who are beyond the typical age for scheduled vaccinations for a three-dose vaccine requires some investment, but school-based scale-up could cost as little as ~$26/girl including the cost of the vaccine and the salaries of existing staff [10]. Studies like these suggest there are feasible, cost-effective options for decreasing cancer-related mortalities in countries where cervical cancer is a major killer.

Finally, Maitland *et al. *[11] offer a closer look at data from the Fluid Expansion as Supportive Therapy (FEAST) trial. The original FEAST study surprised most of us with its finding that fluid boluses are associated with excess mortality at 48 hours in African children with sepsis. To shed some light on the pathophysiologic mechanisms underlying this excess mortality, Maitland *et al*. looked at all-cause mortality by clinical presentation, hemodynamic changes in the first hour and terminal clinical events. The authors hypothesized that excess mortality would be mediated by fluid overload. FEAST continues to surprise us. The excess mortality appears to be related to refractory shock, not fluid overload, suggesting that an ischemia-reperfusion injury following resuscitation may be the problem. Elucidating the mechanism of death in this population of African children has implications for pediatric emergency care measures in all settings, which is further highlighted in a commentary by Simon Finfer and John Myburgh [12]. We seem to have a lot to learn, even about “simple” interventions.

## Competing interests

The author declares that she has no competing interests.

## Author’s information

GB is a neurologist affiliated to Michigan State University and based for half the year in southern Africa. She has a special interest in epilepsy, and especially its management in resource-limited settings. She is also one of the guest editors of our article collection “Medicine for Global Health”.

## Pre-publication history

The pre-publication history for this paper can be accessed here:

http://www.biomedcentral.com/1741-7015/11/72/prepub
